# The impact of preoperative anxiety and education level on long-term mortality after cardiac surgery

**DOI:** 10.1186/1749-8090-7-86

**Published:** 2012-09-14

**Authors:** Zsuzsanna Cserép, Eszter Losoncz, Piroska Balog, Tamás Szili-Török, András Husz, Boglárka Juhász, Miklós D Kertai, János Gál, Andrea Székely

**Affiliations:** 1 Department of Anesthesiology and Intensive Care, Semmelweis University, Budapest, H-1085, Hungary; 2 University of Szeged, Szeged, H-6720, Hungary; 3 Institute of Behavioral Sciences, Semmelweis University, Budapest, H-1085, Hungary; 4 Department of Cardiac Surgery of Gottsegen György Hungarian Institute of Cardiology, Budapest, H-1096, Hungary; 5 Department of Anesthesia and Intensive Care, Gottsegen György Hungarian Institute of Cardiology, Haller u. 29, Budapest, H-1096, Hungary

**Keywords:** Mortality, Cardiac surgery, Anxiety, Depression, Education

## Abstract

**Background:**

Psychosocial factors have shown independent predictive value in the development of cardiovascular diseases. Although there is strong evidence to support the role of psychosocial factors in cardiovascular mortality, there is a scarcity of knowledge about how these factors are related. Therefore, we investigated the relationship between depression, anxiety, education, social isolation and mortality 7.5 years after cardiac surgery.

**Methods:**

After informed consent, 180 patients undergoing cardiac surgery between July 2000 and May 2001 were prospectively enrolled and followed for ten years. During the follow-up period, the patients were contacted annually by mail. Anxiety (Spielberger State-Trait Anxiety Inventory, STAI-S/STAI-T), depression (Beck Depression Inventory, BDI) and the number and reason for rehospitalizations were assessed each year. Those patients who did not respond were contacted by telephone, and national registries were searched for deaths.

**Results:**

During a median follow-up of 7.6 years (25^th^ to 75^th^ percentile, 7.4 to 8.1 years), the mortality rate was 23.6% (95% confidence interval [CI] 17.3-29.9; 42 deaths). In a Cox regression model, the risk factors associated with an increased risk of mortality were a higher EUROSCORE (points; Adjusted Hazard Ratio (AHR):1.30, 95%CI:1.07-1.58)), a higher preoperative STAI-T score (points; AHR:1.06, 95%CI 1.02-1.09), lower education level (school years; AHR:0.86, 95%CI:0.74-0.98), and the occurrence of major adverse cardiac and cerebral events during follow up (AHR:7.24, 95%CI:2.65-19.7). In the postdischarge model, the same risk factors remained.

**Conclusions:**

Our results suggest that the assessment of psychosocial factors, particularly anxiety and education may help identify patients at an increased risk for long-term mortality after cardiac surgery.

## Background

Psychosocial factors such as anxiety, depression, negative affectivity, and social inhibition are measurements that can potentially relate psychological phenomena to the social environment and to pathophysiological changes [1]. Symptoms of anxiety and depression are common psychological disturbances among patients with coronary artery disease, including those undergoing coronary artery bypass graft (CABG) surgery [2], which is an effective therapy for prolonging life and decreasing symptoms in appropriately selected patients [3]. Previously, we showed that patients with high preoperative anxiety have a significantly higher mortality rate four years after surgery [4]. Socially isolated people (e.g., those who are single or have a small social network) have a higher risk for morbidity and mortality according to recent studies [5]. Myocardial infarction-related morbidity and mortality increase with the severity of depressive and anxiety symptoms, especially in patients with a low education status and social support. In the INTERHEART study, low education status markedly increased the incidence of myocardial infarction after adjusting for age, gender, ethnicity, blood cholesterol, hypertension, diabetes and smoking [6].

The aim of the current study was to define the incidence and the time-course of preoperative and postdischarge depression and anxiety in patients underwent cardiac surgery. Furthermore we aimed to examine the association between depression, anxiety scores and mortality 7.5 years after discharge from hospital.

## Methods

### Study population

The study population comprised 197 consecutive patients admitted for elective CABG or valve surgery at the Gottsegen Hungarian Institute of Cardiology between July 2000 and May 2001. There was no eligibility restriction in terms of age, gender, or cardiac condition and no classification of on- and off-pump procedures. After admission to the surgical ward, a study nurse invited the patients to participate in the study. All patients signed an informed consent. The baseline questionnaires were completed 1–5 days before surgery. Seventeen patients (8.6%) were excluded because of a cancelled surgery (*N* = 9) or an inability to complete the psychological tests (*N* = 8). Thus, a total of 180 patients were prospectively studied. The patients who were alive at the end of the 7.5-year follow-up period were considered the survival group, and the patients who died during the follow-up period were considered the non-survival group. The study was approved by the Institutional Medical Ethics Committee. The authors had full access to the data and took responsibility for its integrity. Our previous findings on the fourth year of follow up are reported elsewhere [4].

### Clinical factors

A range of medical and psychosocial factors were assessed as potential determinants of outcome. Medical factors included previous myocardial infarction, previous CABG, history of arrhythmia, congestive heart failure (CHF) (defined as paroxysmal nocturnal dyspnea, dyspnea on exertion, pulmonary congestion on chest x-ray, pedal edema or dyspnea and receiving diuretics or digoxin within the last 2 weeks), diabetes mellitus, hypercholesterolemia, cerebrovascular disease, chronic renal insufficiency, hypertension, and history of psychiatric treatment. Detailed definitions can be accessed on the homepage of the Society of Thoracic Surgeons (STS National Database). The additive EUROSCORE was calculated on the basis of preoperative risk factors to assess and predict the chances of cure and fatal outcome in patients with coronary and heart valve operations [7]. Intraoperative characteristics included the number of grafted vessels, cardiopulmonary bypass (CPB) time, and aortic cross-clamp time. Postoperative complications were defined by the key outcomes of the Society of Thoracic Surgeons Database [8]. After discharge, the occurrence of MACCE (major adverse cardiac and cerebral event) was used to characterize the postdischarge condition of the patient. A MACCE was defined as any of the following: angina; CHF; myocardial infarction; percutaneous coronary angioplasty; survived cardiac arrest; or death due to cardiac causes, stroke, re-CABG, or valve replacement.

### Psychosocial factors

Demographic data on age, gender, living status (alone vs. together with someone), and education (number of successfully finished school years) were collected. Depression was assessed using the Beck Depression Inventory (BDI), a questionnaire previously used with cardiac patients [9]. The BDI is a 21-item self-report instrument measuring the clinical manifestations of depressive symptoms that correspond to the DSM-IV criteria [10]. Cronbach’s alpha in this population was 0.88. Cronbach's alpha is a coefficient of reliability and is commonly used as a measure of the internal consistency or reliability of a psychometric test score. The alpha coefficient ranges in value from 0 to 1 and may be used to describe the reliability of factors extracted from dichotomous and/or multi-point formatted questionnaires or scales (i.e., rating scale: 1 = poor, 5 = excellent). The higher the score is, the more reliable the generated scale [11].

The Spielberger State-Trait Anxiety Inventory state and trait scores (STAI-S and STAI-T) were used to characterize the patients’ anxiety symptoms. The STAI-S measures the transitional emotional status evoked by a stressful situation, such as surgery. The STAI-T score reflects relatively enduring individual differences in the likelihood of anxiety [12]. Cronbach’s alpha in this population was 0.94.

Education was measured by school years and categorized into three groups: less than 8 years of education, more than 12 years of education and more than 8 but less than 12 years. In Hungary, compulsory schooling is finished at the end of the eighth year of school and the upper-secondary education at the end of the twelfth year of school. Education years were calculated as successfully finished school years (i.e., if somebody needed to reattempt the fifth grade, he or she was graded as a fifth grade graduate despite 6 total years of school).

### Follow-up surveillance

The BDI, STAI-S, and STAI-T tests were sent to the patients along with an additional sheet of questions regarding hospitalization and the primary cause of their hospital admission since the last communication. The patients were contacted by mail 6, 12, 24, 36, 48, 60 and 82 months after discharge. For those patients who did not respond and their proxy was unavailable for contact, the Hungarian registry was searched for mortality information at the end of the seventh year.

### End-point assessment

Our end point was all-cause mortality. In 70% of the cases, the clinical data were retrieved from the registry of our hospital where the patients were treated. In the remaining 30%, the data were retrieved from the family doctors' registry. Therefore, the mortality and date of death were available for each patient in the study population.

### Statistical analysis

The data are described as the mean and standard deviation (SD) or the median and interquartile range (25th to 75th percentile) for continuous variables and as the number and percent for categorical variables. The median (25th to 75th percentile) follow-up was computed according to the Kaplan-Meier method. The observation time extended from the date of discharge to the date of death or censoring. Preoperative and operative patient characteristics were compared according to the occurrence of postoperative mortality by means of the Student *t* test or the Mann–Whitney *U* test and the Fisher exact test for continuous and categorical variables, respectively. Paired t-test and Pearson correlation were used for analyzing the pre- and post-discharge BDI and STAI-T points.

The cumulative survival probability and 95% confidence intervals (CIs) were computed and plotted separately for survival and death according to the Kaplan-Meier method. The prognostic value of perioperative and psychosocial factors was evaluated by means of Cox regression. We used Cox proportional-hazards analysis to identify factors associated with mortality after cardiac surgery. Candidate variables were included in the initial Cox regression model if they were associated with mortality in a univariate analysis (P < 0.20). The final multivariable Cox proportional hazards regression model was then derived according to the backward deletion of the least-significant predictors. This statistical model is a type of survival model in which predetermined covariates or risk factors, such as patient characteristics and co-morbidities, are assessed for their independent association with the hazard of mortality. Hazard ratios and the corresponding 95% CIs are reported. Furthermore, we quantified the discriminatory power of the final multivariable model using the c-index, which corresponds to the area under the receiver operating characteristic curve, ranging from 0.5 (performance at chance) to 1.0 (perfect performance). To evaluate the discriminatory power of the final multivariable model, a bootstrap method was used to assess the degree of over-optimism. Over-optimism occurs when the application of statistical modeling techniques results in models that inaccurately predict outcomes for subsequent datasets. A bootstrapping procedure is one method that can be used to try to correct for this over-optimism [13]. The covariates in the final model were fitted for each bootstrap sample. The original dataset was fitted using the coefficients of the bootstrap sample model, and thus, a c-index statistic was generated from this fit on the original dataset. Optimism was then estimated as the difference between the c-index from the bootstrap sample and from the bootstrap model fit on the original sample. All tests were 2 sided, and a value of P < 0.05 was considered to be statistically significant. These analyses were performed using SPSS statistical software (PASW Statistics 18.0; SPSS Inc., Chicago, IL).

In the post-discharge model, only patients who were discharged from the hospital and who had at least one BDI and STAI-T score during the follow up (n = 158) were analyzed.

## Results

During a median follow-up of 7.6 years (25^th^ to 75^th^ percentile, 7.4 to 8.1 years), the mortality rate was 23.6% (95% confidence interval [CI] 17.3-29.9; 42 deaths). Seven patients had in-hospital deaths. Despite the relatively long follow up period, 15 patients did not respond (“were lost to follow-up”) to our questionnaires. Therefore, the analysis of postoperative anxiety and depression levels had a smaller sample size. The non-responder patients were more likely to spend more than two days in the intensive care unit (ICU), and they more frequently had a history of diabetes mellitus compared to those who responded to our follow-up mail questionnaires.

Patients who died on average were older, had a higher risk score (EUROSCORE), and were more frequently diabetic compared to patients who were alive at the end of the follow-up period. Patients who died had also higher preoperative BDI, STAI-S, and STAI-T and higher postdischarge BDI and STAI-T scores and a history of lower education level compared to patients who were alive at the end of the follow-up (Table 1).

**Table 1:** Perioperative characteristics of the study population

	**Survivors(N = 138)**		**Deaths(N = 42)**		
	**Mean/N**	**(SD)/%**	**Mean/N**	**(SD)/%**	**P value**
					
Age (years)	56.9	(10.5)	60.9	(7.9)	0.02
Diabetes	28	20.3%	11	26.2%	0.04
Hypertension	57	41.3%	18	42.9%	0.76
Hyperlipidaemia	54	39.1%	12	28.6%	0.03
Peripheral vascular disease	16	11.6%	9	21.4%	0.09
Arrhythmia	29	21.0%	9	21.4%	0.89
Reoperation	5	3.6%	6	14.3%	0.21
Psychiatric treatment	14	10.1%	12	28.6%	0.003
Anxiolytics/ antidepressants	61	44.2%	12	28.6%	0.02
**Perioperative factors**					
CPB time (min)	72.1	(47.7)	88.2	(46.1)	0.05
EUROSCORE (additive)	3.1	(2.3)	4.5	(2.6)	0.002
ICU stay (days)	1.4	(1.1)	5.4	(11.6)	0.001
Hospital stay (days)	8.9	(3.4)	11.6	(13.8)	0.04
					
BDI	8.7	(5.7)	13.6	(9.8)	<0.001
STAI-S	43.7	(10.8)	48.5	(12.5)	0.02
STAI-T	42.7	(9.2)	51.1	(9.8)	<0.001
School years	11.4	(3.1)	9.5	(2.6)	<0.001
					
MACCE	42	30.4%	36	85.7%	<0.001
Arrhythmia	29	21.0%	9	22.0%	0.90
Malignancy	0	0.0%	4	9.5%	<0.001
Rehospitalization	50	39.7%	22	88.0%	<0.001
BDI (mean)	7.8	(5.5)	14.2	(10.1)	0.004
STAI-T (mean)	40.7	(9.2)	48.5	(12.7)	0.004

**N:** number, **SD:** standard deviation, **CPB time:** cardiopulmonary bypass time, **ICU:** intensive care unit, **BDI**: Beck Depression Inventory, **STAI-S:** state anxiety subscale in the Spielberger State-Trait Anxiety Inventory, **STAI-T:** trait anxiety subscale in the Spielberger State-Trait Anxiety Inventory, **MACCE**: major adverse cardiac and cerebral event.

**Figure 1:**
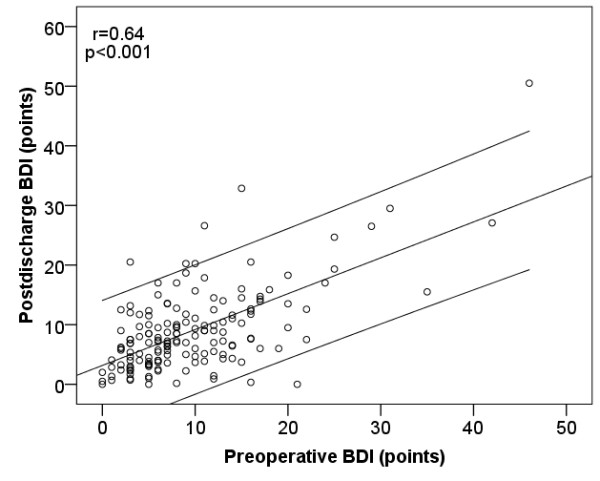
** Figure shows the correlation between the mean of preoperative and postdischarge BDI points.** The line shows correlation and 95% CI.

**Figure 2:**
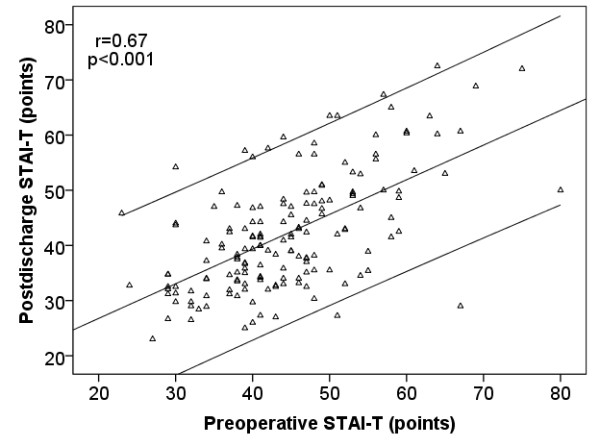
** Figure shows the correlation between the mean of preoperative and postdischarge STAI-T points.** The line shows correlation and 95% CI.

During the follow-up period, the occurrence of a MACCE, malignant disease and rehospitalization were significantly higher in the non-survivor group (Table 1). Preoperative depression (BDI points) and anxiety (STAI T and S scores) were higher in patients who died during the follow-up period (Table 1). There were no significant changes in the mean pre- and postdischarge STAI-T (−0.48 vs. -1.49; p = 0.37) and BDI (−0.83 vs. -0.12; p = 0.58) between patients who died during follow-up and patient who were alive at the end of the follow-up, respectively. Pre- and postoperatively measured BDI (r = 0.64; p < 0.001) and STAI-T (r = 0.67; p < 0.001) scores in the same patients showed a strong correlation (Figures 1 and 2). Patients who died had also a higher preoperative BDI, STAI-S, and STAI-T and higher postdischarge BDI and STAI-T scores: during the follow up the high level of depression and anxiety did not change. We did not find interaction between anxiolytics, psychiatric treatment, BDI and STAI-T values (data not shown).

**Figure 3:**
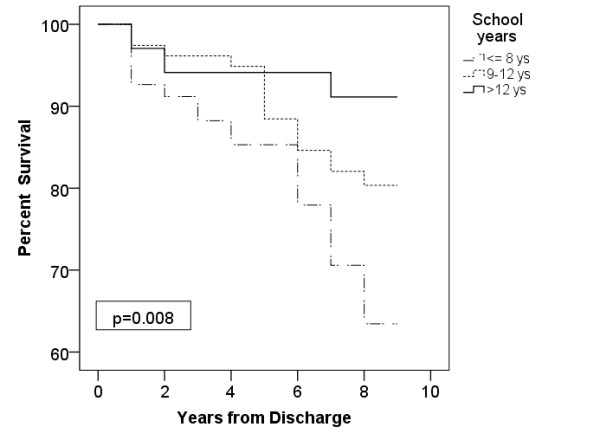
Kaplan-Meier analysis according to the level of education. Time after discharge is plotted against the percent survival.

At the end of follow-up, 64.7% (44 patients) in the low education group, 80.7% (63 patients) of the medium education group and 91.1% (31 patients) of the high education group were alive (Figure 3). Survival was influenced strongly by education. Those patients who had an academic degree had a mean survival time of 8.01 years (95% CI: 7.37 to 8.65), patients with 9 to 12 years of education had a mean survival time of 7.73 years (95% CI: 7.31 to 8.16) and the group with 8 years or less of education had a mean survival time of 7.03 years (95% CI: 6.41 to 7.64). There were significant differences among patients with 8 years or less of education and patients with 8 to 12 years of education (P = 0.032) and patients with an academic degree (P = 0.006) in the survival analysis. Patients with less education had a worse life expectancy. There was no significant difference between patients with 9 to 12 years of education and those with an academic degree (P = 0.18).

In the perioperative and the postdischarge multivariable Cox models, the additive EUROSCORE, length of ICU stay, preoperative STAI-T points, school years and the occurrence of MACCE were independently associated with mortality (Table 2). In the interactive Cox regression model patients were categorized: anxious only, depression only, neither, or both. In the study population, 54 ( 29.8%) patients had no depression or anxiety, 48 (26.5%) patients had only anxiety, (defined as STAI-T ≥40), 14 (7.7%) patients had depression (defined as BDI ≥10) and 65 (35.9%) patients had both anxiety and depression. Compared the survivals and deaths patients with anxiety and anxiety and depression together had significantly higher risk for death (Table 3).

**Table 2:** Adjusted Cox model for medical and psychosocial factors-perioperative and postdischarge model

	**B**	**AHR**	**P value**	**B**	**AHR**	**P value**
		**(95% CI)**			**(95% CI)**	
	**Perioperative model**			**Postdischarge model**		
EUROSCORE	0.222	1.25	0.001	0.264	1.30	0.009
		(1.10-1.41)			(1.07-1.58)	
(Additive, points)						
Preoperative STAI-T (points)	0.054	1.06	0.001	0.055	1.06	0.002
		(1.02-1.09)			(1.02-1.09)	
School years	−0.181	0.83	0.008	−0.156	0.86	0.038
		(0.73-0.95)			(0.74-0.98)	
MACCE	1.946	6.99	0.001	1.980	7.24	<0.001
		(2.99-16.9)			(2.65-19.7)	

In the Cox regression model, perioperative variables p < 0.20 in the univariate analysis were considered. The first model is the perioperative model (second to fourth columns) and the second one is the postdischarge model (fifth to seventh columns). The c-index of the perioperative model was 0.697 before the bootstrapping method and 0.685 thereafter. The c-index of the postdischarge model was 0.691 before the bootstrapping method and 0.681 thereafter. **B:** regression coefficient, **AHR:** adjusted hazard ratio, **STAI-T:** trait anxiety subscale in the Spielberger State-Trait Anxiety Inventory, **MACCE**: major adverse cardiac and cerebral event.

**Table 3:** Interactive Cox regression model for medical factors, anxiety and depression

	**B**	**AHR**	**(95,0% CI)**		**P value**
Euroscore	0.166	1.18	(1.05	1.33)	0.007
School years	−0.191	0.83	(0.72	0.95)	0.008
MACCE	2.057	7.82	(3.02	20.27	<0.001
Anxiety	1.401	4.06	(1.16	14.20)	0.028
Depression	−11.28	0.82	(0.42	1.63)	0.98
Anxiety + Depression	1.59	4.93	(1.45	16.68)	0.01

In the Cox regression model, perioperative variables p < 0.20 in the univariate analysis were considered. The c-index of the model was 0.697 before the bootstrapping method and 0.685 thereafter. **B:** regression coefficient, **AHR:** adjusted hazard ratio, **MACCE**: major adverse cardiac and cerebral event.

## Discussion

We found that trait scores of anxiety (STAI-T), and education level were associated with a higher risk of mortality after adjusting for medical factors and postdischarge major cardiac events during 7.5 years of follow up after cardiac surgery.

Psychosocial risk factors, such as low socio-economic status, chronic family or work stress, social isolation, negative emotions (e.g., chronic depression or acute anxiety), and negative personality patterns, such as Type-D-pattern or hostility, have been shown to be significantly associated with the development of coronary artery disease and with the occurrence of adverse outcome in patients with established coronary artery disease [14].

Numerous studies have underlined the importance of preoperative depression and anxiety for mortality after cardiac surgery [2,15,16]. Although anxiety and depression are highly co-morbid and tend to share risk factors, anxiety is a discrete emotional experience. Anxiety has been characterized as a future-oriented, negative affective state with a component of fear, resulting from the perception of threat and the individual’s perceived inability to predict, control, or obtain the desired results in upcoming situations. Symptoms of anxiety may be adversely associated with a high risk of ischemic heart disease [17], and anxiety has been associated with an increased risk of myocardial infarction and fatal ischemic heart disease after CABG [16].

Depression is a prevalent comorbidity in patients with coronary artery disease. The prevalence ranges from 14–47% with higher rates seen most often in patients with unstable angina or those awaiting CABG surgery [2]. Depression on the day before surgery and depression that persists months after surgery were associated with a two- to threefold increased risk of mortality [18]. According to a recent study about women at high risk for cardiovascular disease not only the presence of depression but also the severity of depression influenced the outcome jointly with or independent of known risk factors [19]. Further research is needed to find the factors responsible for constant high STAI-T and BDI scores. On the other hand, survivors were more frequently treated than those who died during the follow-up.

In a parallel analysis of preoperative anxiety and depression, only anxiety was significantly associated with increased mortality after adjusting for known mortality risk factors [20]. We have used the additive EUROSCORE for risk estimation for cardiac surgery [7]. The addition of anxiety and education to the risk model might help to further refine the risk factors associated with increased mortality. We found that preoperative STAI-T scores were associated with increased 7-year mortality, and these results highlight the importance of preoperative screening for anxiety in routine clinical practice. In a similar study, patients who had major depression in the hospital just before discharge were more than twice likely to die or be readmitted for cardiac causes in the 12 months after discharge from the hospital than those without this disorder. They also found that major depressive disorder increased the frequency of cardiac events independent of the usual risk factors [15]. These findings suggest that a surgically successful cardiac operation does not always correlate with the improvement of individual life expectancies after surgery [2] and does not decrease level of depressive symptoms [21].

The overlap between anxiety and depression has long been discussed. Most recent research on the pathophysiological links between negative emotions and ischemic heart disease has revealed that depression and anxiety have several similar effects on coronary events, including increased catecholamine levels, indicators of autonomic dysfunction (increased heart rate, decreased heart rate variability, and decreased baroreceptor sensitivity), increased platelet activity, and subacute chronic inflammation [22]. Low education and income are important determinants of all-cause mortality and cardiovascular mortality [23] among patients with myocardial infarction. Low income and education are related to a higher risk profile and poorer treatment [24]. In accordance, in our study, a higher level of education was associated with a longer survival time. Patients with a high level of education are likely to have a higher income and therefore can afford the more expensive “healthy” diet and sport activities [23].

The limitation of our study is the small sample size and thus the lower statistical power. Nevertheless, the applied statistical method strengthened the results of this study. However, it was a single center study; hence, the results may not be generalizable to similar patient populations. Furthermore, we have excluded patients who could not fill out our questionnaires and those undergoing emergent surgery or a concomitant procedure, suggesting that our results may not necessarily generalize to higher risk patients. Smoking history, actual blood pressure and medication after discharge were not recorded during the follow-up period. Additionally, we cannot exclude the possibility that the data for the non-responders could have changed the results. The strengths of this study are that in addition to anxiety, education has also been evaluated. Also, this study has a long-term follow up, and the data were collected prospectively. The model created and validated in this study can have an important clinical impact, as it provides a preoperative estimate of an individual patient’s risk of death. We have enrolled patients undergoing CABG and/or valve surgery, which can also influence our results, although a previous report did not find significant in-hospital differences in mortality and morbidity [25].

## Conclusion

Our results indicate the long term impact of preoperative anxiety, and lower education for mortality after cardiac surgery. However, patients with cardiovascular disease and treated depression had modest improvement in depressive symptoms but no improvement in cardiac outcomes. Therefore, further research is needed about mechanisms by which anxiety, education, or other psychosocial factors may be related to objective health endpoints [26].

## Abbreviations

STAI-S: State anxiety subscale in the Spielberger state-trait anxiety inventory; STAI-T: Trait anxiety subscale in the Spielberger state-trait anxiety inventory; BDI: Beck depression inventory; CI: Confidence interval; AHR: Adjusted hazard ratio; CABG: Coronary artery bypass graft; CBP: Cardiopulmonary bypass; MACCE: Major adverse cardiac and cerebral event; CHF: Congestive heart failure; ICU: Intensive care unit.

## Competing interests

The authors declare that they have no competing interests.

## Authors’ contributions

ASZ, PB and MDK designed the study. ASZ, ZCS, AH and EL collected the clinical data. ASZ, ZCS, TSZT analysed and interpreted the data. ASZ and ZCS drafted the manuscript. ASZ, JG, TSZT and MDK made critical revision of the manuscript for important intellectual content. ASZ and ZCS performed the statistical analysis. JB performed the operation. All authors read and approved the final manuscript.
